# Bleaching efficacy and reduction of cytotoxicity of 6% H₂O₂ in-office bleaching gels formulated with hyaluronic acid, NF_TiO₂ nanoparticles, and violet LED irradiation: in vitro study

**DOI:** 10.1007/s10266-025-01292-1

**Published:** 2026-01-06

**Authors:** Priscila Borges Gobbo de Melo, Marcos Roberto de Lima Benati, Matheus Kury, Rafael Antônio de Oliveira Ribeiro, Carlos Alberto de Souza Costa, Fernando Luis Esteban Florez, Vanessa Cavalli

**Affiliations:** 1 Department of Restorative Dentistry, Piracicaba Dental School, University of Campinas, Avenida Limeira, 901, Piracicaba, SP Brazil; 2 Dental Research Division, School of Dentistry, Paulista University (UNIP), São Paulo, SP Brazil; 3 Department of Dental Materials and Prosthodontics, School of Dentistry, São Paulo State University (UNESP), Araraquara, SP Brazil; 4 Department of Physiology and Pathology, School of Dentistry, São Paulo State University (UNESP), Araraquara, SP Brazil; 5 Department of Restorative Sciences, University of Oklahoma - Health Sciences Center, Oklahoma City, OK USA

**Keywords:** Tooth bleaching, Hydrogen peroxide, Hyaluronic acid, Titanium, Violet LED

## Abstract

The objective is to evaluate the bleaching efficacy and cytotoxicity of innovative low-concentration in-office gels containing 6% hydrogen peroxide (H₂O₂), hyaluronic acid (HA), or carbomer 940 (CAR) as thickeners, titanium dioxide nanoparticles co-doped with nitrogen and fluorine (NF_TiO₂) and irradiated with violet LED. Bovine enamel/dentin disks adapted to artificial pulp chambers were randomly assigned to seven groups (*n* = 8): NC (negative control), HP35 (commercial control), HA, HA-HP6 + LED, HA-HP6-NP + LED, CAR-HP6 + LED, and CAR-HP6-NP + LED. The bleaching protocol consisted of three 30-min sessions conducted at 7-day intervals, each session involving 20 violet LED cycles (1-min activation / 30-s pause). Bleaching efficacy was assessed by measuring color change (ΔE₀₀) and whiteness index (ΔWI_D_). Additionally, hydrogen peroxide diffusion, cell viability, oxidative stress, and cell morphology were evaluated. One-way ANOVA was used for statistical analysis, followed by Games-Howell post-hoc test (ΔE_00_ and ΔWI_D_) and Tukey’s test (cytotoxicity) (α = 0.05). Experimental gels showed ΔE₀₀ and ΔWI_D_ values comparable to those of HP35, with no significant differences observed. The incorporation of NF_TiO₂ (HA-HP6-NP + LED and CAR-HP6-NP + LED) significantly reduced H₂O₂ diffusion and oxidative stress while increasing cell viability (*p* < 0.05); therefore, preserving cell morphology. The HA-containing group exhibited greater cell proliferation. HP35 demonstrated higher cytotoxicity and cell deformations. Experimental gels containing NF_TiO₂ and 6% H₂O₂, formulated with either HA or CAR as thickeners, exhibited bleaching efficacy comparable to HP35, along with a significant reduction in H₂O₂ diffusion and oxidative stress. The incorporation of nanoparticles significantly enhanced cell viability. The experimental gels may reduce pulpal cytotoxicity and H₂O₂ diffusion, potentially lowering pulp damage and tooth sensitivity. Further studies are required to assess their effects on enamel integrity.

## Introduction

In-office dental bleaching is widely used in dentistry due to its ability to provide a rapid and effective change in tooth color [1–3]. This technique involves the application of bleaching gels containing high concentrations of hydrogen peroxide (H₂O₂; HP; 25–40%) [4, 5]. The oxidative action of H₂O₂ is responsible for degrading chromophores present in enamel and dentin [5]. Despite its efficacy, residual H₂O₂ that is not fully degraded during the process can diffuse through dentinal tubules, reaching the pulp tissue and causing structural damage [1, 6–9].

Among the limitations associated with in-office bleaching, tooth sensitivity stands out as the most frequently reported adverse effect, with a prevalence exceeding 50% [7]. The intensity of sensitivity is influenced by factors such as H₂O₂ concentration, application time, and tooth structure, including enamel and dentin thickness [10, 11]. This adverse effect is linked to the trans-amelodentinal diffusion of H₂O₂ and reactive oxygen species (ROS), which, upon reaching pulp cells, induce oxidative stress, inflammatory responses, and, in severe cases, partial pulp necrosis [6, 8, 9]. The greater the amount of H₂O₂ reaching the pulp, the higher the oxidative stress level, leading to cellular damage and inflammation of this specialized connective tissue [11–15].

To mitigate the adverse effects of bleaching without compromising its efficacy, various strategies have been proposed. These include the use of gels with lower H₂O₂ concentrations and the incorporation of catalysts in bleaching gels to prevent excessive pulp exposure to H₂O₂, which could cause severe tissue damage and, consequently, increase sensitivity [8, 16–18]. The catalysis of H₂O₂ accelerates its decomposition into highly reactive byproducts, reducing hydroxyl radical formation, linked to sensitivity, while enhancing bleaching efficacy. Additionally, due to the short half-life of H₂O₂, the risk of pulp cell damage is minimized [19–21].

Recent technological advancements include the use of doped nanoparticles (NP), such as nitrogen (N) and fluoride (F)-modified titanium dioxide (NF_TiO₂) [22–24], which act as catalysts in bleaching gels activated by violet light (405–410 nm) [8, 18, 25]. The co-doping process modifies the electronic structure of TiO₂, allowing these nanoparticles to absorb visible light efficiently. When irradiated with violet light, they enhance the generation of reactive oxygen species (ROS), particularly superoxide radicals (O₂⁻), which are highly effective in breaking down chromogenic compounds, while simultaneously reducing the formation and diffusion of more cytotoxic molecules toward the dental pulp. This photochemical mechanism enables the use of low-concentration hydrogen peroxide (H₂O₂) gels, promoting effective bleaching while significantly minimizing adverse effects such as tooth sensitivity [8, 16].

Furthermore, the use of biopolymers such as hyaluronic acid (HA) has gained prominence in the medical field. Its viscoelastic properties, biocompatibility, and ability to create a favorable molecular environment offer significant advantages over traditional synthetic polymers, such as carbomer 940 (CAR) [26–28]. When used as a thickening agent in bleaching gels, HA demonstrates excellent physicochemical properties, contributing to the stability of the formulation. Moreover, due to its bioactive characteristics, hyaluronic acid may support cell proliferation and viability, particularly of pulp cells, acting not only to preserve the physical properties of dental enamel but also to promote a safer and more biologically compatible environment during bleaching procedures [29–32].

Given the need to improve bleaching safety and efficacy, and considering the synergistic potential of these components, the present study aims to evaluate the effects of experimental bleaching gels containing HA as a thickener, NF_TiO₂ nanoparticles, 6% hydrogen peroxide, and violet LED irradiation, for bleaching efficacy, H₂O₂ diffusion, MDPC-23 cell viability, oxidative stress, and pulp cell morphology using Scanning Electron Microscopy (SEM). The null hypotheses established were that the experimental gels: (1) would not exhibit the same bleaching efficacy and (2) would not reduce the cytotoxicity of MDPC-23 pulp cells compared to the commercial bleaching gel containing 35% hydrogen peroxide.

## Materials and methods

### Sample size calculation and experimental design

The sample size calculation was defined based on evidence from previously published studies of recognized relevance that employed similar methodologies for bleaching efficacy and cytotoxicity analyses. The estimated sample size of eight specimens per group (*n* = 8) was defined for all response variables [8, 19, 31]. Each protocol was performed in two separate experiments (duplicates) to minimize systematic errors and ensure reproducibility of the observations.

All experimental procedures were performed by a single trained and calibrated operator to ensure consistency and reliability throughout the study. The analyses, including data collection and statistical evaluation, were conducted by a separate specialized operator under blinded conditions, who was unaware of the treatment allocation for each group. This approach was implemented to minimize potential risk of bias and enhance the accuracy and reproducibility of the results.

### Synthesis of NF_TiO₂ nanoparticles

The NF_TiO₂ nanoparticles were synthesized via a solvothermal method following previously established protocols [22, 24]. Initially, a solution was prepared using 1.7 g of titanium (IV) butoxide (Sigma-Aldrich, 97%), 4.6 g of ethanol (Decon Labs), 6.8 g of oleylamine (Sigma-Aldrich, 70%), 7.1 g of oleic acid (Sigma-Aldrich, 90%), and 5% ammonium fluoride (based on crystalline Ti content; ACS, Alfa Aesar). Subsequently, 20 mL of 4% hydrated alcohol (18-MΩ Milli-Q, Decon Labs) was added to this mixture. The solution was transferred to a high-pressure reactor system and maintained at 180 °C for 24 h under magnetic stirring. After cooling, the supernatant was removed, and the solid was washed three times with anhydrous ethanol to eliminate residual surfactants. The purified TiO₂ nanoparticles were stored in ethanol and centrifuged (8000 rpm, 15 min).

### Preparation of experimental gels

Two different polymers were used as thickeners in the bleaching gels. The first was carbomer 940, a commercially available synthetic hydrophilic polymer (Carbomer 940 NF, Spectrum, Gardena, CA), prepared according to the protocol described by Kury et al. (2022) [18]. In brief, the polymer was dispersed in ultrapure water using an autonomous planetary and orbital mixer (Speed Mixer, DAC 400.1 FVZ, FlackTek Inc., Laudrum, SC, USA).

The second polymer used was hyaluronic acid (Sigma-Aldrich Chemical, St. Louis, MO, USA), a hydrophilic biopolymer. For its preparation, 395.3 g of ultrapure water and 4.8 g of phenoxyethanol were weighed, to which 8 g of powdered hyaluronic acid was added. The mixture was then homogenized in a mixer (Speed Mixer, Landrum, SC, USA) until the desired viscosity was achieved.

The NF_TiO_2_ nanoparticles (~ 40 mg/mL), previously suspended in ethanol, were prepared for incorporation into the gels. For this, 1 mL of the suspension was transferred to a conical centrifuge tube and centrifuged at 8000 rpm for 5 min. After removing the ethanol, the NPs were incorporated into 20 g of each thickener. This step was performed using a mixer (Speed Mixer, DAC Iso.1 FVZ, FlackTek Inc., Laudrum, SC, USA) at 2450 rpm for 20 s, resulting in gels with a final NP concentration of 5% and a pH close to 6.0 [32]. Finally, the 6% HP solution was prepared from a standard 35% HP solution (Sigma-Aldrich, St. Louis, MI, USA).

### Sample preparation and selection

Bovine incisors with intact enamel, free of cracks and fractures, were selected and stored at 4 °C in a 0.5% thymol solution (Labsynth, Diadema, SP, Brazil) for disinfection for up to 30 days. Fifty-six enamel–dentin disks (*n* = 8/group) were obtained from the middle third of the buccal surface of each incisor using a diamond glass drill (⌀8 mm, Di Martino Brocas Diamantadas Ltda, Campinas, SP, Brazil) attached to a benchtop drill (Pratika FSB16P, Schultz, Joinville, SC, Brazil). Each disc had an approximate diameter of 5 mm, and its enamel–dentin thickness was standardized at 2.3 mm ± 0.2 mm through mechanical polishing (AROTEC, São Paulo, Brazil) [31, 33].

The dentin surface was initially flattened with #600 grit silicon carbide sandpaper to ensure parallelism and standardization. Subsequently, the enamel surface was progressively polished using silicon carbide sandpapers (#600, 800, 1200; 3 M Brazil, Sumaré, SP, Brazil) and a felt disk with a diamond suspension (1 μm and ½ μm abrasive particles) for 1 min [31].

### Group distribution

The dentin region of the samples was isolated with a clear enamel coating, and the samples were immersed in a neutral pH (7.0) black tea solution (Dr. Oetker, São Paulo, SP, Brazil) for 24 h. They were then stored in artificial saliva containing 1.5 mM calcium (CaCl₂), 0.9 mM phosphate (NaH₂PO₄), 0.15 mM potassium (KCl), and a 0.1 mol/L Tris buffer, adjusted to pH 7.0, for 7 days to stabilize the coloration [31, 32]. After this period, the enamel surface was cleaned using pumice (SS White, São Cristóvão, RJ, Brazil) and water to remove superficial pigments.

Subsequently, the disks were systematically positioned on a white surface to allow colorimetric readings using a portable spectrophotometer (Easy Shade, Vita Zahnfabrik, Bad Säckingen, Germany). The values of the color coordinates (L*, a*, b*, h, and C) obtained from the readings were used to allocate the specimens into seven groups randomly. A one-way ANOVA test confirmed the absence of significant differences among groups concerning the color coordinates (*p* > 0.05). The groups were then randomly assigned to each proposed bleaching protocol, as described in Table 1.

**Table 1:** Group distribution

Group	Treatments
NC	Negative control – no treatment
HA	Hyaluronic acid hydrogel (without H_2_O_2_)
HP35	Commercial bleaching gel (35% H_2_O_2,_ Whiteness HP, FGM, Joinville, SC, Brazil)
HA-HP6 + LED	Experimental gel with hyaluronic acid as a thickener, 6% hydrogen peroxide, and violet LED irradiation
HA-HP6-NP + LED	Experimental gel with hyaluronic acid as a thickener, 6% hydrogen peroxide, 5% NF_TiO_2_ nanoparticles, and violet LED irradiation
CAR-HP6 + LED	Experimental gel with carbomer 940 as a thickener, 6% hydrogen peroxide, and violet LED irradiation
CAR-HP6-NP + LED	Experimental gel with carbomer 940 as a thickener, 6% hydrogen peroxide, 5% NF_TiO_2_ nanoparticles, and violet LED irradiation

### Bleaching procedure

The gels, with and without NF_TiO_2_ incorporation, were manually prepared by adding a 6% HP solution in a 2:1 ratio (%HP: thickener) [18]. During the first bleaching session (T_1_), 20 μL of either commercial or experimental gel was applied to the enamel surface using a viscosity pipette. The HP35 gel remained in contact with the surface for 30 min without renewal. The experimental gels were irradiated with violet LED light (Bright Max Whitening, MMOptics, São Carlos, SP, Brazil) in cycles of 1-min irradiation, interspersed with 30-s intervals, maintaining the light tip at an 8 mm distance from the enamel surface, corresponding to 30 min of bleaching (irradiation + interval). The violet LED used in this study exhibits a peak wavelength of 401.82 nm, a total power output of 1.202 W, and a total radiant emittance of 137.4 mW/cm^2^ at the sample surface, ensuring consistent experimental conditions [25]. The same procedures were repeated for the three sessions, which were performed at 7-day intervals. Between each session, specimens were stored at 37 °C in artificial saliva, remaining under the same conditions for 14 days (T_1_) after the last bleaching session.

### Evaluation of bleaching efficacy (ΔE_00_ and ΔWI_D_)

At time points T_0_ (before bleaching) and T_4_ (14 days after the last bleaching session), enamel-dentin disks were positioned on a white and opaque surface to allow color coordinate measurements using a digital spectrophotometer Easyshade V (Vita Zahnfabrik, Bad Säckingen, Germany). For each measurement, three readings were performed on each sample in different positions on the same surface, rotating the specimen between each reading, and the average value was calculated. All measurements were conducted under standardized lighting conditions to minimize variability and improve reliability. The color variation (ΔE_00_, Eq. 1) was calculated based on the CIEDE2000 system equation, considering the L*, a*, b*, h, and C values obtained at T_0_ and T_4_ [34–37]. The change in whiteness was assessed using the Whiteness Index for Dentistry (WI_D_, Eq. 2), with ΔWI_D_ determined as the difference between WI_D_ values at T_1_ and T_0_ (T_1_– T_0_). The adopted ΔE_00_ thresholds for perceptibility (PT) and acceptability (AT) were 0.8 (PT) and 1.8 (AT) units, respectively [35]. The adopted ΔWI_D_ thresholds for PT and AT were 0.72 (PT) and 2.6 (AT) units, respectively [36].

1 $$\Delta {\mathrm{E}}_{00} = \left[ {\left( {\Delta {\mathrm{L}}^{\prime } {\mathrm{/K}}_{{\mathrm{L}}} {\mathrm{S}}_{{\mathrm{L}}} } \right)^{2} + \left( {\Delta {\mathrm{C}}^{\prime } {\mathrm{/K}}_{{\mathrm{C}}} {\mathrm{S}}_{{\mathrm{C}}} } \right)^{2} + \left( {\Delta {\mathrm{C}}^{\prime } {\mathrm{/K}}_{{\mathrm{H}}} {\mathrm{S}}_{{\mathrm{H}}} } \right)^{2} + {\mathrm{R}}_{{\mathrm{T}}} *\left( {\Delta {\mathrm{C}}^{\prime } {\mathrm{/K}}_{{\mathrm{C}}} {\mathrm{S}}_{{\mathrm{C}}} } \right)*\left( {\Delta {\mathrm{H}}^{\prime } {\mathrm{/K}}_{{\mathrm{H}}} {\mathrm{S}}_{{\mathrm{H}}} } \right)} \right]^{1/2}$$

2 $${\mathrm{WI}}_{{\mathrm{D}}} = 0.511{\mathrm{L}} * { - 2}{\mathrm{.324}}^{{\mathrm{a*}}} - 1.100{\mathrm{b*}}$$

## Experimental protocol

### Cell culture

MDPC-23 cells, an immortalized odontoblast-like cell line derived from the dental papilla of mouse molars, stored in liquid nitrogen at the Laboratory of Experimental Pathology and Biomaterials at the Araraquara School of Dentistry – UNESP, were thawed and cultured in 75 cm^2^ flasks (KASVI, São José dos Pinhais, PR, Brazil). Cell passages were performed every three days until an adequate number of cells was obtained for this stage of the research. The pulp cells were then plated in 96- and 24-well plates (KASVI, Curitiba, PR, Brazil) and cultivated in Dulbecco’s Modified Eagle’s Medium (DMEM; Gibco, Grand Island, NY, USA) supplemented with 10% fetal bovine serum (FBS; Gibco), 100 IU/mL penicillin, 100 µg/mL streptomycin (Gibco), and 2 mmol/L glutamine (Gibco). The cultures were maintained in a humidified atmosphere at 37°C, with 5% CO₂ and 95% air. Cells were counted using an inverted light microscope and replicated as necessary to ensure sufficient numbers for the experiments [6].

### Experimental procedure

**Fig. 1:**
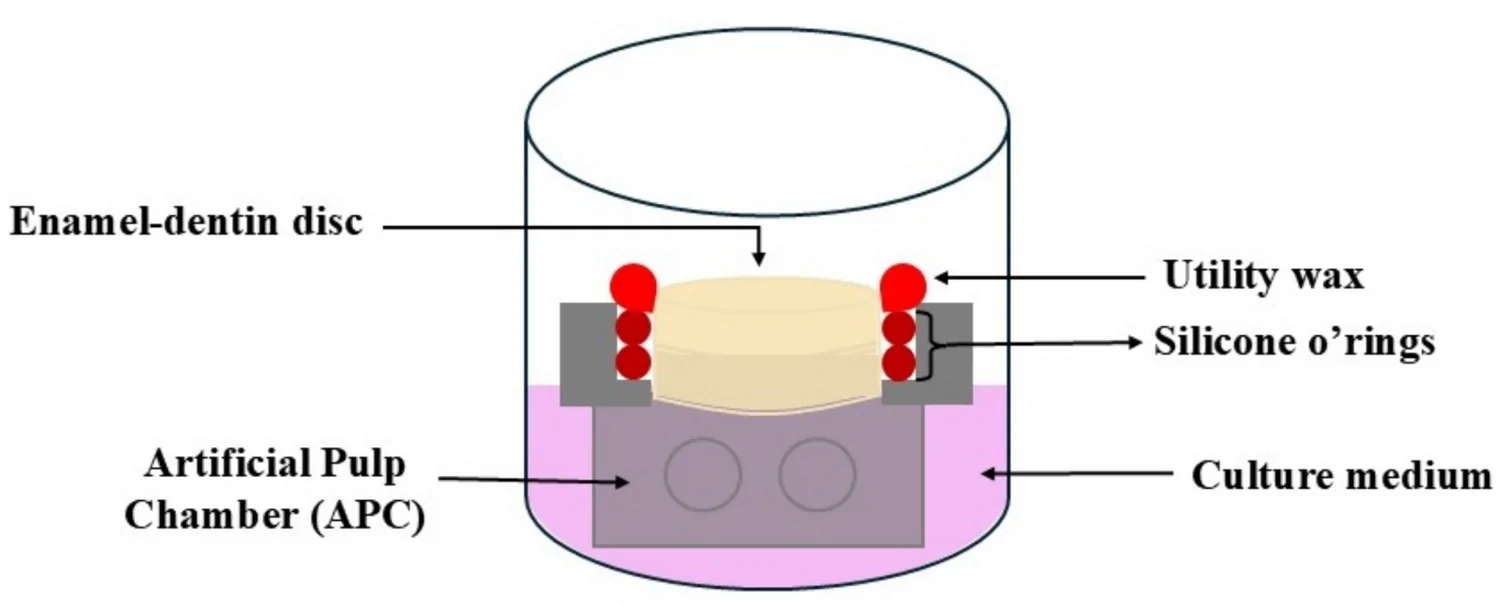
Representative diagram of the disk/APC assembly positioned in one of the wells of a sterile 24-well acrylic plate. The enamel-dentin disk is held in place within the APC by two silicone rings, while its edge is sealed with utility wax

Fifty-six enamel-dentin disks (n = 8) were adapted into artificial pulp chambers (APCs) using two silicone rings, sealed with utility wax (Cera 7 Rosa Wilson; Polidental, Cotia, SP, Brazil) to prevent the infiltration of the bleaching gel into the pulp chamber (Fig. 1). The disc/APC sets were sterilized with ethylene oxide (Acecil; Central de Esterilização Comércio e Indústria, Campinas, SP, Brazil) and then placed in 24-well plates (KASVI) containing 1 mL of DMEM without FBS to allow the solution to remain in contact with the exposed dentin and enamel, either receiving or not receiving the bleaching treatments [11–15].

Immediately after the bleaching procedures, the extracts, consisting of DMEM and the bleaching gel components that had diffused through the enamel-dentin disks, were collected, homogenized, and aliquoted into 100 μL portions for subsequent testing [6].

### Quantification of H₂O₂ in the extracts

To quantify H₂O₂ diffusion through the enamel–dentin disks, a 100-μL aliquot of each extract was combined with 900 μL of 2 mol/L acetate buffer (pH 4.5) in 24- or 96-well plates. Then, 500 μL of this solution was transferred to tubes containing 100 μL of leuco crystal violet reagent (0.5 mg/mL; Sigma-Aldrich, St. Louis, MI, USA), 50 μL of horseradish peroxidase (1 mg/mL; Sigma-Aldrich), and 2750 μL of distilled water. Absorbance was measured using a spectrophotometer (λ = 596 nm, Synergy H1, Biotek Instruments, Winooski, VT, USA). Optical density values were converted into micrograms of H₂O₂ per mL of extract using a standard curve derived from known H₂O₂ concentrations. The results were expressed as a percentage, with the HP35 set as the reference for 100% residual H₂O₂ diffusion [11–15].

## Cytotoxicity analysis

### Cell viability

For this assay, a 10% Alamar Blue solution (Life Technologies, Grand Island, NY, USA) was prepared in DMEM without FBS. Then, 500 μL of this solution was added to each well containing the extracts, and the plates were incubated in a 5% CO₂ atmosphere at 37 °C for 4 h. In its oxidized form, Alamar Blue exhibits a blue color, which is converted to a reduced (pink) form due to mitochondrial activity. Subsequently, 200 μL of each sample (extract) was transferred to a 96-well plate, and mitochondrial activity was determined by measuring fluorescence of the reduced dye using a spectrophotometer (excitation: 530–560 nm; emission: 590 nm, Synergy H1). The mean absorbance value of the negative control group was set as 100% cell viability, and this value was used to determine viability across all groups [11–15].

### Oxidative stress

To assess intracellular ROS, cells cultured in 96-well plates (KASVI) were pre-treated with the fluorescent probe carboxy-H₂DCFDA (Invitrogen, San Francisco, CA, USA) at 5 mM for 30 min at 37 °C before being exposed to 100 μL of the extracts. Following exposure, fluorescence was immediately measured (excitation: 492 nm, emission: 518 nm, Synergy H1, Biotek Instruments). The obtained values were compared to the mean of the NC group, which was used as a reference for data normalization [11–15].

### Cell morphology

Cells were exposed to the collected extracts and remained adhered to glass slides placed in 24-well plates (*n* = 2/group). The samples were then fixed in 2.5% glutaraldehyde for 60 min (Sigma-Aldrich) and post-fixed in 1% osmium tetroxide (Sigma-Aldrich) for the same duration. Subsequently, the samples underwent gradual dehydration using an ethanol series (30, 50, 70, 90, and 100%) and were treated with HMDS (1,1,1,3,3,3-hexamethyldisilazane, Sigma-Aldrich). After being placed in a desiccator for at least 72 h, the slides were mounted on metal stubs using carbon adhesive tape (Koch Electron Microscopy Ltda., São Paulo, SP, Brazil), sputter-coated with gold, and analyzed using SEM (FEI Inspect S 50, USA) [31], at magnifications of 1000 × and 4000x.

### Statistical analysis

Quantitative data were tabulated and subjected to normality (Shapiro–Wilk, *p* > 0.05) and variance homogeneity (Levene, *p* > 0.05) tests. For color analyses (ΔE_00_ and ΔWI_D_), a one-way analysis of variance (ANOVA) with Welch’s correction was applied, followed by Games-Howell post-hoc testing due to variance heterogeneity. For other tests (H₂O₂ quantification, cell viability, and oxidative stress), assumptions were met, allowing the application of one-way ANOVA followed by Tukey’s test. Statistical analyses were conducted using SPSS software (version 26.0, IBM, Chicago, IL, USA) and GraphPad Prism (version 9.0, GraphPad, San Diego, CA, USA), with a significance level of 5%.

## Results

### Evaluation of bleaching efficacy (ΔE_00_ and ΔWI_D_)

**Fig. 2:**
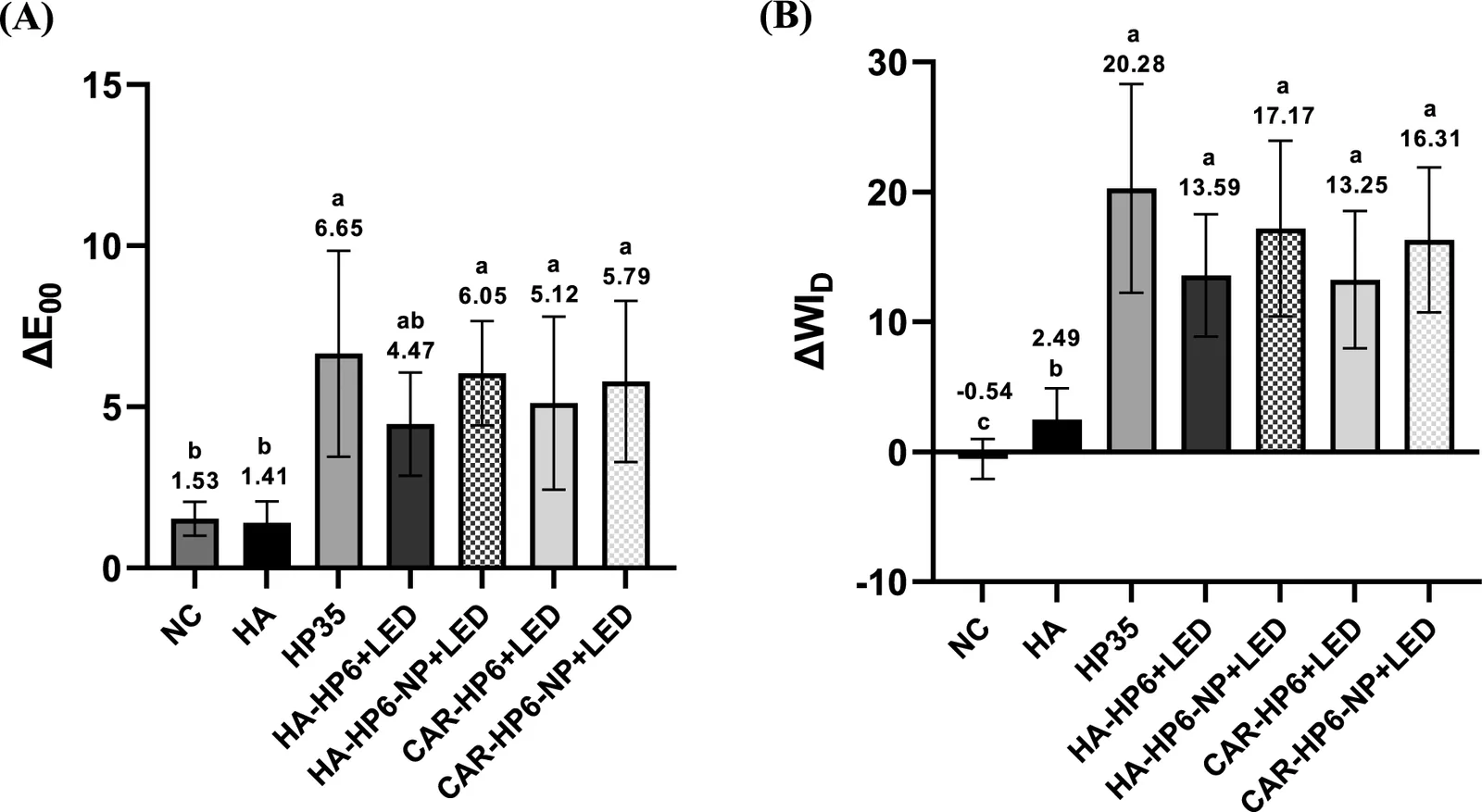
Analysis of bleaching efficacy, ΔE_00_ (**A**) and ΔWI_D_ (**B**). Bars represent mean values and standard deviations of color change. Numbers indicate the mean values for each group. Different letters denote statistically significant differences (ANOVA–Welch/Tukey; *p* < 0.05; *n* = 8)

In the ΔE_00_ analysis (Fig. 2A), the NC and HA groups showed no statistically significant difference, both presenting the lowest mean bleaching efficacy (*p* = 1.00). The HA-HP6 + LED group did not differ significantly from either the lower-efficacy groups (NC and HA) or the higher ΔE_00_ groups (*p* > 0.05). In contrast, the HP35, HA-HP6-NP + LED, CAR-HP6 + LED, and CAR-HP6-NP + LED groups presented the highest ΔE_00_ values, with no statistically significant differences observed among them (*p* > 0.05).

The ΔWI_D_ results (Fig. 2B) indicated no statistical difference in bleaching potential between the experimental groups and the HP35 group (*p* > 0.99). However, when compared to the NC and HA groups, a significant difference was observed.

For ΔE₀₀, the overall effect size was large (η^2^ = 0.50), with the highest means observed in HP35 (6.65; 95% CI = 4.25–9.06), HA-HP6-NP + LED, and CAR-HP6 + LED (6.05; 95% CI = 4.60–7.49), and the lowest in HA (1.41; 95% CI = 0.65–2.17) and NC (1.53; 95% CI = 1.05–2.00), indicating statistically and clinically meaningful differences. For ΔWI_D_, the overall effect was even greater (η^2^ = 0.65; ω^2^ = 0.60), with post hoc comparisons showing lower values for HA and NC, while the remaining groups exhibited higher values. Individual pairwise effect sizes (η^2^ = 0.58–0.72; 95% CI = 0.24–0.84) further underscore the robustness and consistency of the results, highlighting the strong influence of the treatments on bleaching efficacy.

### Quantification of H₂O₂ in the extracts

**Fig. 3:**
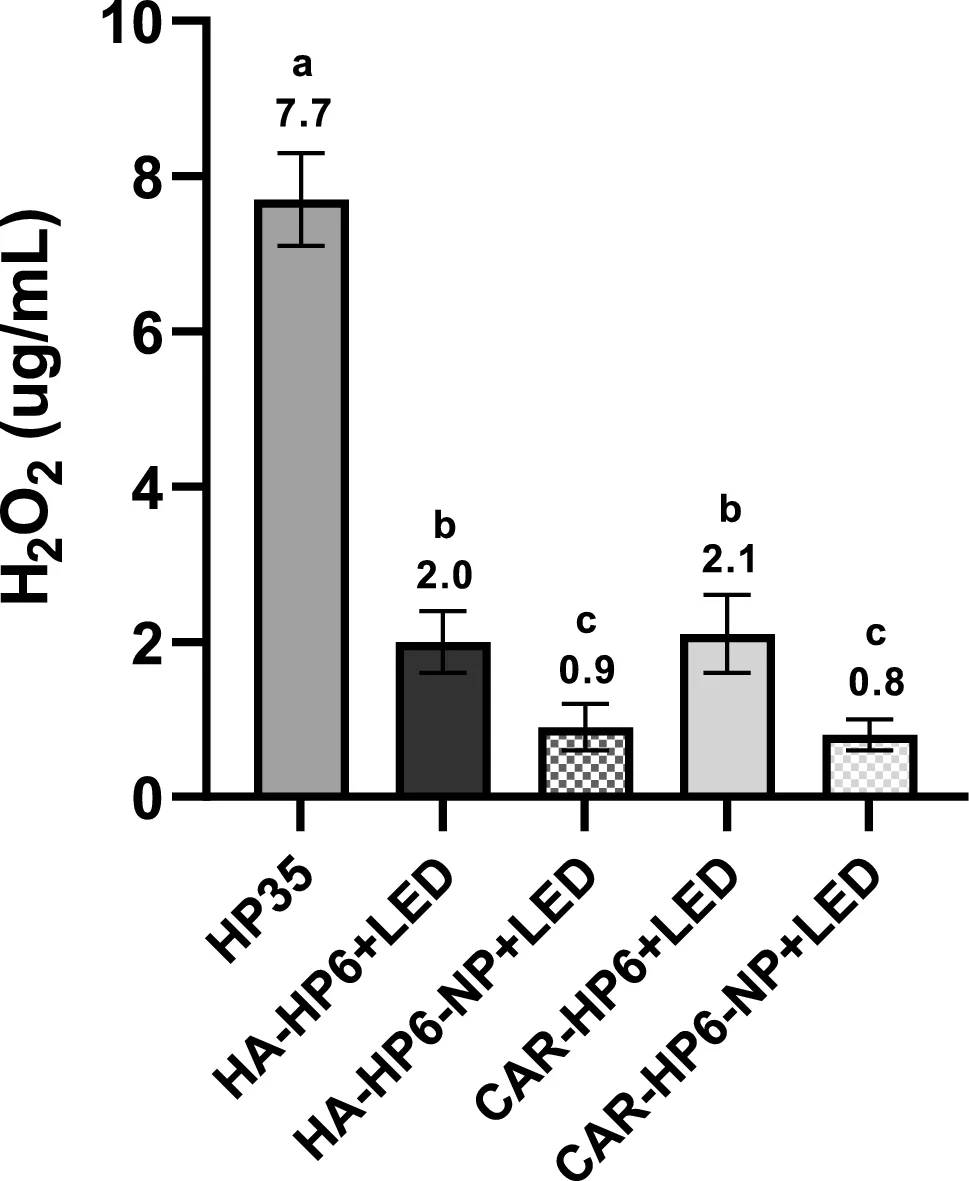
Analysis of H₂O₂ diffusion. Bars represent mean values and standard deviations. Numbers indicate the mean values for each group. Different letters denote statistically significant differences (ANOVA/Tukey; *p* < 0.05; *n* = 8)

Figure 3 presents the trans-amelodentinal diffusion values of H₂O₂ (μg/mL) after the first bleaching session. The HP35 group exhibited the highest H₂O₂ diffusion values, being statistically superior (*p* < 0.05) to all other groups. In the experimental groups, regardless of the presence of NP, the amount of diffused H₂O₂ was significantly lower than in the HP35 group (*p* < 0.001). However, it is noteworthy that the NP-containing groups demonstrated the lowest mean H₂O₂ diffusion values.

The overall effect size for diffusion was extremely large (η^2^ = 0.977; *f* = 6.51), with the highest mean observed in HP35 (1.85; 95% CI = 1.62–2.08). The lowest diffusion values were recorded in HA-HP6-NP + LED (0.98; 95% CI = 0.85–1.12) and CAR-HP6 + LED (0.87; 95% CI = 0.74–0.97), reflecting statistically significant differences.

### Cytotoxicity analysis

**Fig. 4:**
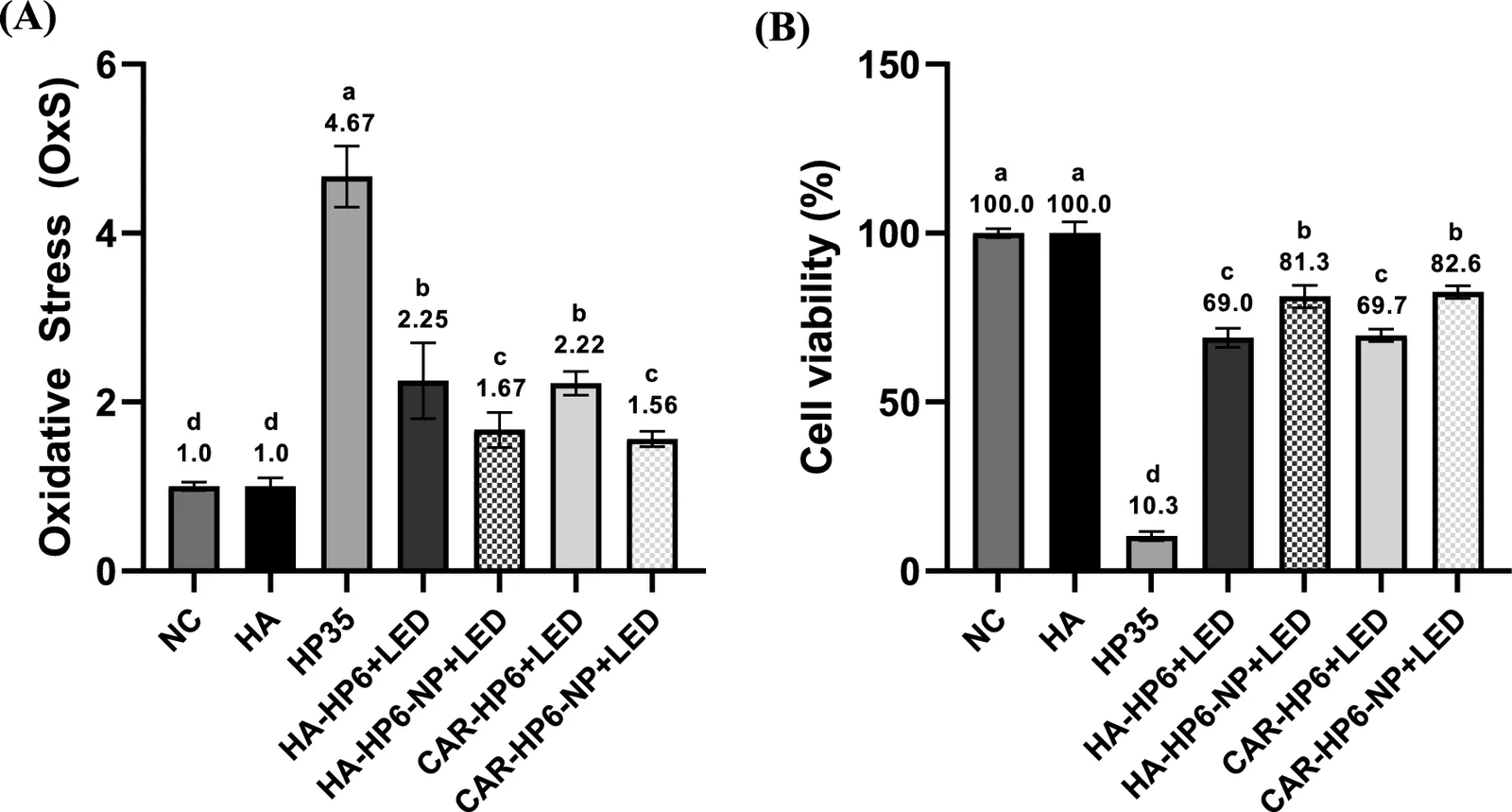
Oxidative stress (**A**) and cell viability analysis (**B**). Bars represent mean values and standard deviations. Numbers indicate the mean values for each group. Different letters denote statistically significant differences (ANOVA/Tukey; *p* < 0.05; *n* = 8)

As shown in Fig. 4 (A and B), the NC and HA groups demonstrated 100% cell viability, whereas the HP35 group exhibited the lowest viability indices among the analyzed groups (*p* < 0.001). The gels containing HP 6%, both with HA and CAR, showed lower cell viability than the NC and HA groups but significantly higher than the HP35 group (*p* < 0.001). The addition of NP to the bleaching gels resulted in a significant increase in cell viability (*p* < 0.001) and a reduction in oxidative stress levels (*p* < 0.001).

Oxidative stress levels exhibited an extremely high overall effect size (η^2^ = 0.96). The highest means were observed in HP35 (4.67; 95% CI = 4.37–4.97), followed by HA-HP6 + LED (2.25; 95% CI = 1.87–2.63) and HA-HP6 + LED (2.22; 95% CI = 2.11–2.34), whereas the lowest values occurred in NC (1.00; 95% CI = 0.96–1.04) and HA (1.00; 95% CI = 0.91–1.08). Pairwise comparisons using Tukey’s post hoc test revealed large to extremely large effect sizes (Cohen’s *d* = 2.3–15.0; 95% CI = 1.3–19.2), confirming the strong and consistent influence of the treatments on oxidative stress induction.

Cell viability also showed an extremely high overall effect size (η^2^ = 0.99). The highest viability was observed in NC and HA (99.99; 95% CI = 98.91–101.07 and 97.21–102.76, respectively), followed by HA-HP6-NP + LED (81.34; 95% CI = 78.56–84.12) and HA-HP6-NP + LED (82.65; 95% CI = 81.06–84.24). The lowest viability was recorded in HP35 (10.34; 95% CI = 9.23–11.45). Pairwise comparisons using Tukey’s post hoc test revealed large to extremely large effect sizes (Cohen’s *d* = 3.7–68.5; 95% CI = 2.8–72.1), highlighting the strong impact of treatment composition and the cytoprotective role of hyaluronic acid and NF_TiO₂ nanoparticles.

### Cell morphology

**Fig. 5:**
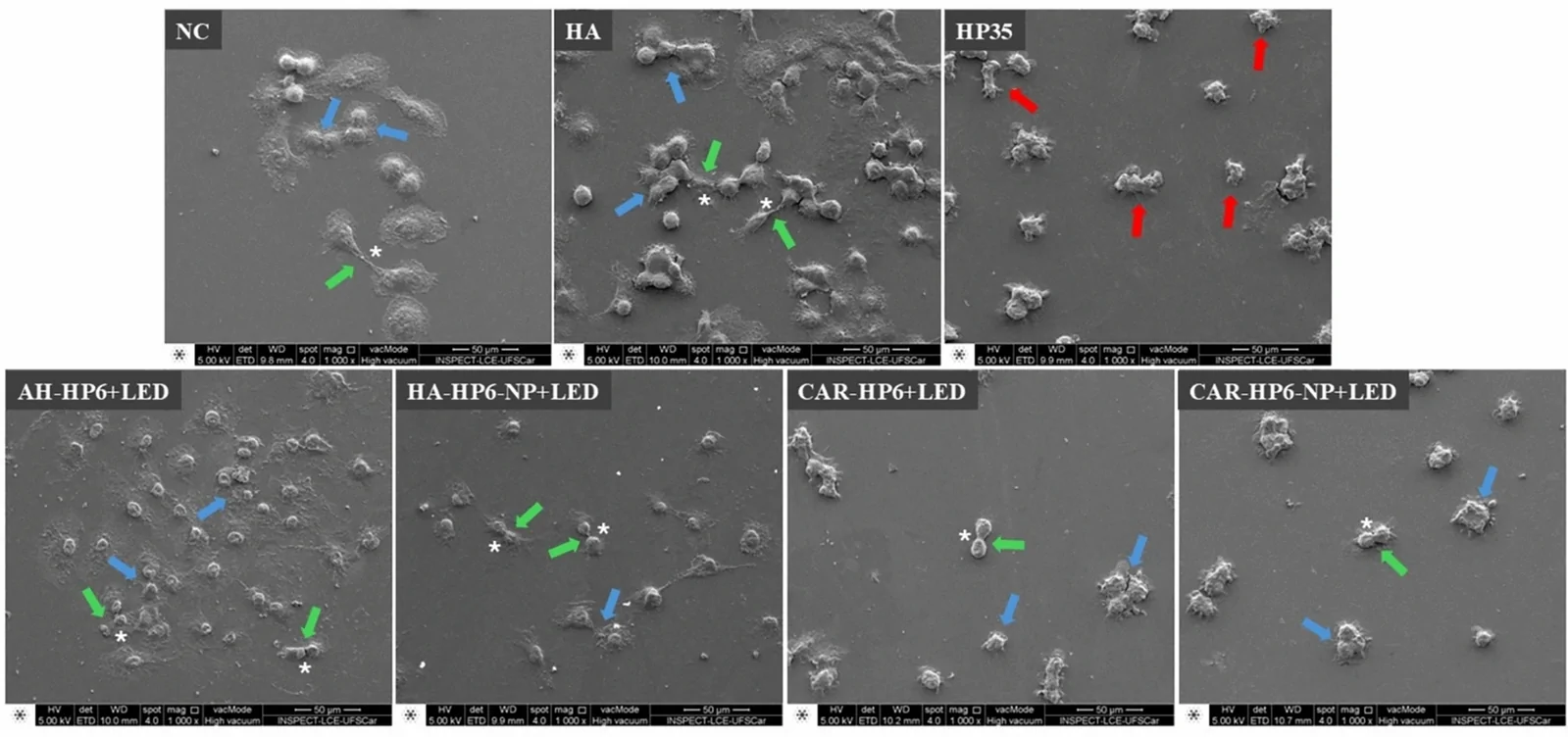
Representative SEM images (1000 × magnification) showing glass slides where MDPC-23 cells were cultured and subsequently exposed to extracts collected from the APCs after the bleaching protocols. Blue arrows indicate cells with an extensive cytoplasm and spherical morphology; green arrows point to membrane and cytoplasmic extensions of cells undergoing mitosis (*); red arrows indicate cells with significant morphological alterations

**Fig. 6:**
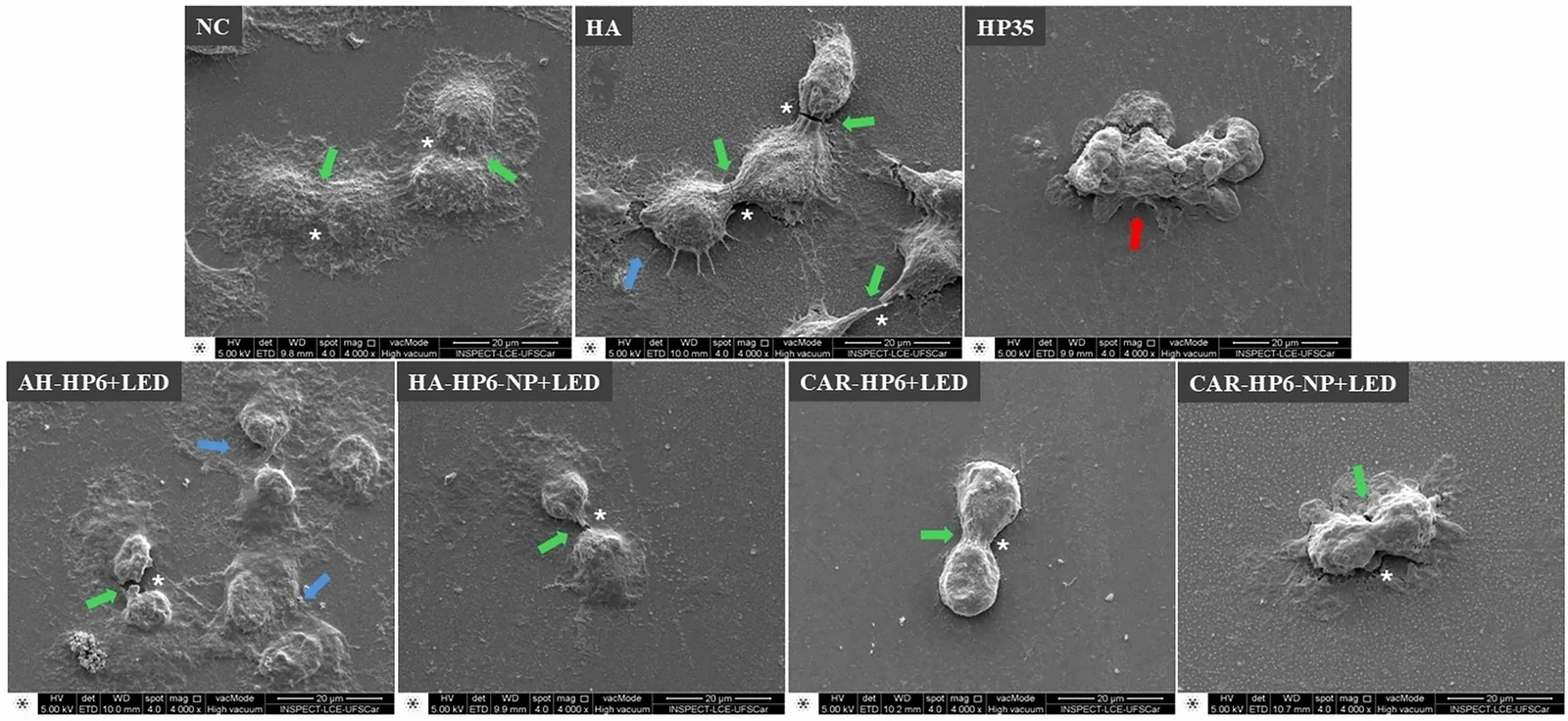
Representative SEM images (4000 × magnification) showing glass slides where MDPC-23 cells were cultured and subsequently exposed to extracts collected from the APCs after the bleaching protocols. Blue arrows indicate cells with an extensive cytoplasm and spherical morphology; green arrows point to membrane and cytoplasmic extensions of cells undergoing mitosis (*); red arrows indicate cells with significant morphological alterations

Figures 5, 6 present the cell morphology analyzed via SEM following the application of different bleaching protocols. In the NC and HA groups, a greater number of MDPC-23 cells adhered to the substrate, with the HA group showing the highest cellular proliferation. In the experimental groups, although a reduction in the number of adherent cells was observed compared to the NC and HA groups, the morphology of the remaining cells remained similar to that of the NC and HA groups. Under these conditions, the cells exhibited broad membranes and pronounced cytoplasmic extensions, characteristic indicators of mitosis.

Conversely, in the HP35 group, significant deformation of the adherent cells was observed, differing from all other analyzed groups. Blue arrows indicate cells with an extensive cytoplasm and spherical morphology; green arrows point to membrane and cytoplasmic extensions of cells undergoing mitosis (*); red arrows indicate cells with significant morphological alterations.

## Discussion

The findings of this study provide valuable insights into the use of experimental bleaching gels containing hyaluronic acid (HA) and NF_TiO₂, highlighting their bleaching efficacy and biological effects compared to a high-concentration in-office bleaching gel (35% H₂O₂). The results confirm that the incorporation of NP and the use of lower concentrations of H₂O₂ can mitigate adverse effects specifically related to pulp cytotoxicity, such as cell viability, oxidative stress, cell morphological changes, and diffusion of H₂O₂ into the pulp chamber, without compromising the efficacy of bleaching [8, 31].

The experimental groups that utilized NF_TiO₂ combined with violet LED irradiation (HA-HP6-NP + LED and CAR-HP6-NP + LED) exhibited ΔE₀₀ and ΔWI_D_ values comparable to those of the commercial gel containing 35% H₂O₂ (HP35). Consequently, the first null hypothesis was rejected, reinforcing the theory that NF_TiO₂ nanoparticles act as catalysts, enhancing the bleaching action of H₂O₂ even at reduced concentrations. The violet LED (λ = 405 nm ± 15 nm) further promoted ROS generation, facilitating efficient chromophore degradation and significant tooth color alteration [18, 25].

These results are consistent with those reported by Kury et al. (2022) [18], who demonstrated that bleaching gels containing NF_TiO₂ NPs combined with 6% H₂O₂ achieved outcomes comparable to high-concentration formulations, despite using only one-fifth of the peroxide concentration. Moreover, the PT and AT thresholds observed in the experimental groups exceeded previously reported values [34–37], even in formulations without NP incorporation.

The superior performance of the NF_TiO₂ NPs was attributed to their high absorption within the violet light range (390–420 nm) [38], showing double the activity of commercial TiO₂ nanoparticles (P25, Degussa) [22]. This enhancement was linked to co-doping with nitrogen and fluoride, which increased photocatalytic activity and promoted more efficient H₂O₂ decomposition into stable and long-lasting ROS [8, 32], essential for oxidizing long-chain chromophores responsible for dentin discoloration [5, 25, 31].

Furthermore, properties reported in previous studies, such as the predominance of the anatase phase, uniform pore size distribution, and high catalytic efficiency, may have contributed to the performance of the nanoparticles [18, 22]. Previous studies highlight that violet light irradiation alone can enhance the esthetic outcomes of low concentration bleaching gels [8, 9, 18], primarily due to an increase in gel temperature and the accelerated decomposition of H₂O₂ into short-lived ROS [8]. In this context, the combination of NF_TiO₂ NPs with violet LED exhibited a synergistic effect, significantly improving the efficacy of bleaching gels containing 6% H₂O₂. These findings emphasize the importance of combining light activation with NPs to enhance the esthetic outcomes of in-office dental bleaching [8].

Higher H₂O₂ concentrations resulted in greater trans-amelodentinal diffusion, increased oxidative stress, and a significant reduction in cell viability. Thus, the second null hypothesis, which stated that the experimental gels would not reduce MDPC-23 pulp cell cytotoxicity compared to the commercial bleaching gel (HP35), was rejected. In the experimental groups, lower H₂O₂ diffusion was observed through the enamel-dentin disks. These data confirm that the amount of H₂O₂ diffused into the culture medium is directly related to its concentration in the bleaching gel [8, 11, 31]. Furthermore, it is important to highlight that diffusion was further reduced with the addition of NF_TiO₂ NPs (HA-HP6-NP + LED and CAR-HP6-NP + LED).

The significant reduction in trans-amelodentinal H₂O₂ diffusion observed with the experimental gels represents a major biological advantage over HP35. This effect can be attributed to the controlled release of H₂O₂ facilitated by HA and the catalytically accelerated decomposition promoted by NF_TiO₂ NPs. This combination reduces the amount of residual H₂O₂ capable of diffusing, making it a key feature in preventing pulp tissue damage, a common adverse effect associated with bleaching treatments.

The HP35 group, which used 35% H₂O₂, induced greater oxidative stress and significantly reduced cell viability, highlighting the aggressiveness of this concentration on pulp cells. In contrast, gels containing 6% H₂O₂ combined with NF_TiO₂ NPs demonstrated lower H₂O₂ diffusion, reducing cytotoxicity in MDPC-23 cells. This reduction may be attributed to the higher catalytic efficiency of NPs, which accelerated H₂O₂ decomposition, promoting its dissociation into ROS [8, 20, 21]. These ROS effectively degrade chromophores in enamel and dentin while minimizing the formation of hydroxyl radicals, which are often associated with increased tooth sensitivity [19–21]. Additionally, the presence of HA may have contributed to a more biocompatible environment, further reducing cytotoxicity.

SEM revealed that the groups treated with experimental bleaching gels showed a slight reduction in the number of adherent cells compared to the negative controls yet maintained preserved cell morphology. The cells displayed regular contours, predominantly spherical shapes, smooth surfaces, and intact volumes, features indicative of viable and metabolically active cells. These observations suggest low cytotoxicity and indicate that the presence of HA may support cellular integrity, likely due to its hydrophilic nature, ability to retain water, and formation of a hydrated matrix that promotes cell adhesion and proliferation.

Conversely, cells in the group treated with the HP35 exhibited marked morphological alterations, including shrinkage, irregular membrane surfaces, and collapsed structures, consistent with severe cytotoxic damage and potential loss of viability. These findings corroborate those of de Benati et al. [31], who observed similarly preserved morphology and indicators of proliferative activity, such as expanded cytoplasmic regions and well-defined cell boundaries, following treatment with the same HA-based experimental gels. Collectively, the results reinforce the cytoprotective effect of HA against oxidative damage induced by hydrogen peroxide, particularly when combined with photocatalytic nanoparticles and violet LED irradiation, which appear to mitigate cytotoxic effects while preserving cellular morphology.

Although the present study was conducted in vitro, the clinical use of the violet LED should strictly follow the manufacturer’s guidelines and be performed under the indication and supervision of a qualified professional. The use of protective eyewear is essential for both the patient and the operator to prevent direct exposure to violet radiation and to minimize potential ocular damage. Furthermore, maintaining a controlled distance of 8 mm between the light source and the oral tissues is crucial to reduce possible adverse effects, thereby ensuring greater safety and efficacy during the procedure.

The combination of low concentration H₂O₂ with catalysts and biocompatible polymers, together with the use of photocatalyst nanoparticles, presents a significant potential to improve the patient experience by reducing tooth sensitivity and achieving esthetic outcomes comparable to those obtained with high-concentration gels. However, despite these promising results, certain limitations must be considered for an appropriate interpretation. As an in vitro model was used, direct extrapolation to clinical conditions is limited, since the in vivo oral environment involves additional complexities, including salivary flow, pH variations, biofilm presence, immune responses, tissue regeneration, and cellular and vascular interactions within the pulp, which cannot be fully simulated in laboratory settings. Future in vivo studies are necessary to validate the safety and efficacy of bleaching gels containing NF_TiO₂ nanoparticles under clinically relevant conditions, allowing a more comprehensive assessment of long-term effects and confirming the biocompatibility of the materials. Accordingly, the advancements proposed in this study open new possibilities for the development of safer and more effective bleaching gels for dental applications.

## Conclusions

Within the limitations of this study, the following conclusions can be drawn:

- The experimental bleaching gels containing hyaluronic acid as a thickener, 5% NF_TiO₂ nanoparticles, and violet LED irradiation demonstrated bleaching efficacy comparable to that of the high-concentration commercial bleaching gels (HP35).
- The incorporation of NF_TiO₂ nanoparticles into the experimental bleaching gels significantly reduced H₂O₂ diffusion and oxidative stress while enhancing cell viability, resulting in lower pulp cytotoxicity.

## Data Availability

The datasets generated and analyzed during the current study are available from the corresponding author on reasonable request.
